# Acupuncture is associated with a positive effect on odour discrimination in patients with postinfectious smell loss—a controlled prospective study

**DOI:** 10.1007/s00405-021-06872-9

**Published:** 2021-05-25

**Authors:** Tanja Drews, Thomas Hummel, Bettina Rochlitzer, Bettina Hauswald, Antje Hähner

**Affiliations:** 1 Department of Otorhinolaryngology, Smell and Taste Clinic, Technische Universität Dresden, Technical University of Dresden Medical School, Fetscherstrasse 74, 01307 Dresden, Germany; 2 Department of Otorhinolaryngology, Bundeswehrkrankenhaus Berlin, Scharnhorststr 13, 10115 Berlin, Germany; 3 Klinik Für Augenheilkunde, Städtisches Klinikum Görlitz, Girbigsdorfer Str 1-3, Görlitz, Germany

**Keywords:** Acupuncture, Smell loss, Olfaction, Sniffin’ sticks

## Abstract

**Introduction:**

Smell disorders are common in the general population and occur e.g., after infections, trauma or idiopathically Treatment strategies for smell loss range from surgery, medication to olfactory training, depending on the pathology, but they are limited This study examined the effect of acupuncture on olfactory function.

**Methods:**

Sixty patients with smell loss following infections of the upper respiratory tract were included in this investigation Half of the study group were randomly assigned to verum acupuncture and the other half to sham acupuncture Olfaction was measured by means of the “Sniffin’ Sticks” test battery (odour threshold, discrimination and identification).

**Results:**

Compared to sham acupuncture, verum was associated with an improvement of smell function as measured by the TDI score (*p* = 0.039) The improvement was largely determined by improvement in odour discrimination, and was significantly better in patients with a shorter duration of the disorder.

**Conclusion:**

The present results suggest that acupuncture is an effective supplementary treatment option for patients with olfactory loss.

## Introduction

Olfactory impairment is common in the general population and results in a loss of quality of life [1, 2]. While there are several valid and reliable tools available for the diagnosis of olfactory loss, the treatment possibilities of olfactory dysfunction are limited Causes of smell disorders include sinunasal diseases, acute infections of the upper respiratory tract, head trauma, neurodegenerative diseases, medication including chemotherapy, environmental factors and ageing [3–5]. In effect, approximately 5% of the population are estimated to exhibit functional anosmia with the prevalence being highest in the older population [6, 7].

Treatment strategies for smell loss are related to the cause of the disorder Particularly effective therapies are available for chronic inflammation of the upper airway system including surgery or topical or systemic anti-inflammatory medication [8, 9]. Interestingly, in clinical routine topical steroids are used irrespective of the aetiology [10]. Another effective method in treating various forms of smell loss is olfactory training [11]. Systematic, repeated exposure to odours may lead to an improvement of olfactory function in patients with post-infectious, posttraumatic and neurodegenerative smell loss [11–15]. Nevertheless, apart from these treatment options, therapies for olfactory loss are relatively poorly developed [16, 17].

Hence, the present study focuses on the treatment of patients with postinfectious smell loss who represent one of the largest groups of patients with olfactory disorders with about 18–45% of patients presenting themselves with smell loss [1]. Postinfectious smell loss is caused by an infection of the upper respiratory tract (URTI) that persists after the infection has passed [18]. The exact pathological mechanism is unclear but its possible lesion sites include damage to the sustentacular cells or the olfactory receptor neurons in the mucosa or the olfactory bulb [19, 20]. URTI-associated smell loss typically occurs after the fifth decade of life and is seen more frequently in women than in men [18]. About 25% of these patients describe parosmia, possibly due to the partial loss of olfactory receptors [21]. In addition to olfactory training, treatment strategies include vitamin A nasal drops or systemic alpha-lipoic acid [22, 23].

Sporadically, acupuncture has been used in postinfectious olfactory loss [24]. Acupuncture is an important part of traditional Chinese medicine Those that use needle acupuncture believe that every living being is filled with the energy “Qi” that flows within the body along meridians on which the individual acupuncture points are located Diseases are explained by disruptions in the flow of Qi [25, 26].

The needling of acupuncture points has been shown to be associated with analgesic and relaxing effects It is, therefore, not only positive for the body but similarly for the mental state of the patient Needling stimulates nerves, which results in an activation of the central nervous system [27]. The procedure involves the application of 10–20 needles to certain cutaneous points, where they remain for about 30 min [28]. The points are determined by palpation The procedure is repeated on different days There should be a noticeable effect, for example in the form of improvement of symptoms, after about 8 sessions on different days [29]. Side effects are rare, with only about 3% of treated patients describing pain, local infections and hematoma [28].

So far there are only a few studies, that will now be described, that have examined the effect of acupuncture on smell disorders in a controlled way However, previous results already indicate a positive effect of this approach In the study by Vent et al., 15 patients with postinfectious smell loss were treated with acupuncture and compared to 15 patients who had been treated with Vitamin B After 10 weeks of acupuncture treatment, there was a significant improvement in smell function in the acupuncture group compared to the Vitamin B group [30]. However, the significance of this study was questioned later [31]. A non-blinded control condition was used in a study by Dai et al who reported olfactory improvement in postinfectious patients after acupuncture compared to a patient group without treatment [32]. Hauswald et al applied acupuncture in the context of a non-randomized, non-controlled study in a larger group of patients with various aetiologies of smell loss [33]. They reported a significant improvement in olfactory function, especially in postinfectious smell loss Anzinger used laser acupuncture on healthy subjects in a double-blinded single-application approach and found a positive, acute effect on olfaction which was measured using the Sniffin’ Sticks Test [34]. Furthermore, in a case report smell improvement in one patient receiving acupuncture treatment was reported, this was based, however, on self-assessment [35].

The aim of this single-centre, prospective, placebo-controlled, patient-blinded study was to investigate the change of olfactory function in patients with postinfectious smell loss following 12 acupuncture sessions, twice per week, each 30 min long Based on previous reports, we expected that acupuncture has a positive effect on smell function in postinfectious patients.

## Methods

### Patients

Sixty subjects were recruited consecutively between August 2012 and February 2013 at the Smell and Taste Clinic of the Department of Otorhinolaryngology of the TU Dresden All patients had received the diagnosis of postinfectious smell loss following a detailed, structured history and a full otorhinolaryngological examination including nasal endoscopy [36]. Inclusion and exclusion criteria are listed in Table 1 All participants provided written informed consent The study was approved by the Ethics Committee of the Medical Faculty of the TU Dresden (ethics approval number EK 78,032,012).

**Table 1:** Inclusion and exclusion criteria

Inclusion criteria	Exclusion criteria
Smell loss directly following an URTI	Symptom-free interval
18 years of age or older	URTI during the study period
Written informed consent	Neurological diseases that may impair the sense of smell (e.g Parkinson’s or Alzheimer’s disease)
Absence of chronic infection of the nasal cavities or sinuses	Dermatological problems that may complicate the application of the needles

### Olfactory testing

The “Sniffin’ Sticks” test battery was applied before and after treatment involving tests for odour threshold, odour discrimination and odour identification [37]. The sum of the scores from the three subtests resulted in the TDI-score (Threshold, Discrimination and Identification).

### Acupuncture treatment

Subjects were randomly attributed to treatments so that one half received verum acupuncture and the other half received sham acupuncture, sham meaning that fake points, rather than actual acupuncture points were needled Both groups underwent acupuncture 12 times with approximately 2 sessions per week The acupuncture as well as the sham acupuncture consisted of needling specific points using sterile acupuncture needles This was performed by the same person in every session, and in every session the same points were needled.

After completion of the study treatment, patients who had received the placebo condition were offered the verum acupuncture following the conclusion of the study.

**Image 1:**
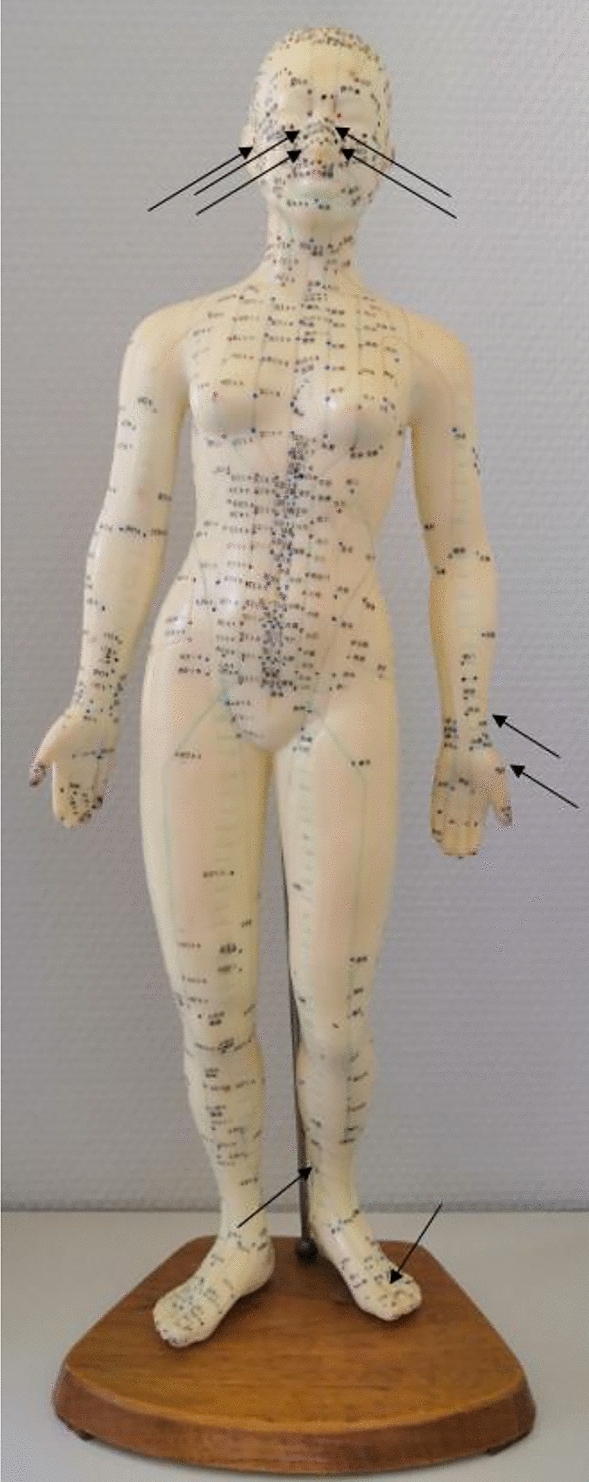
Verum-points on face and ear (Bl 3, LG23, Di 20, NP 12, Op 16), on the arm (Di 4, Lu 7) and on the leg (MP 6, Ma 44)

The points chosen for the verum acupuncture were comparable to those used in previous studies and can be seen in Image 1 [30, 33]. All points were used symmetrically on both sides of the body.

The points used in the sham acupuncture were the same points used in the ACUSAR- study [38]. The points used are listed in Table 2.

**Table 2:** Verum and placebo acupuncture points

Location	Acupuncture Points	Points for sham acupuncture
Head	Bladder 3	/
	Governing Vessel 23	/
	Large intestine 20	/
	Extra point 12	/
Ear	Ear 16	/
Arm	Lung 7	Deltoideus
	Large intestine 4	Upper Arm
Leg	Spleen 6	Thigh 1
	Stomach 44	Thigh 2
Back	/	Back 1
	/	Back 2

### Statistical analysis

The data were analysed using SPSS 27.0 (SPSS Inc., Chicago, Ill, USA) If not mentioned otherwise, all data are shown as means ± standard deviation (SD) or numbers (%), significance level was set at *p* < 0.05 (two-tailed test) Pearson statistics were used for correlational analyses.

## Results

Sixty patients, 23 males and 37 females were included in the study The verum group was comprised of 17 hyposmic patients and 13 functionally anosmic patients, the sham acupuncture group of 2 normosmic, 14 hyposmic and 14 anosmic patients All patients completed the study without any exception.

No adverse effects were encountered during the acupuncture sessions Descriptive statistics of the patient groups at baseline are shown in Table 3.

**Table 3:** descriptive statistics at baseline before treatment; TDI: summated score from odour threshold, discrimination, and identification; standard deviation in brackets

	Verum acupuncture	Sham acupuncture	*p*-value
Age in years	63.0 (13.6)	66.3 (10.1)	0.29
duration of smell loss in years	3.9 (4.3)	5.1 (5.3)	0.34
TDI score	17.12 (5.62)	17.47 (6.99)	0.86
Odour Threshold	2.08 (1.68)	2.29 (1.79)	0.84
Odour Discrimination	8.37 (2.53)	8.03 (2.99)	0.75
Odour Identification	6.67 (2.83)	7.13 (3.57)	0.60

**Fig 1:**
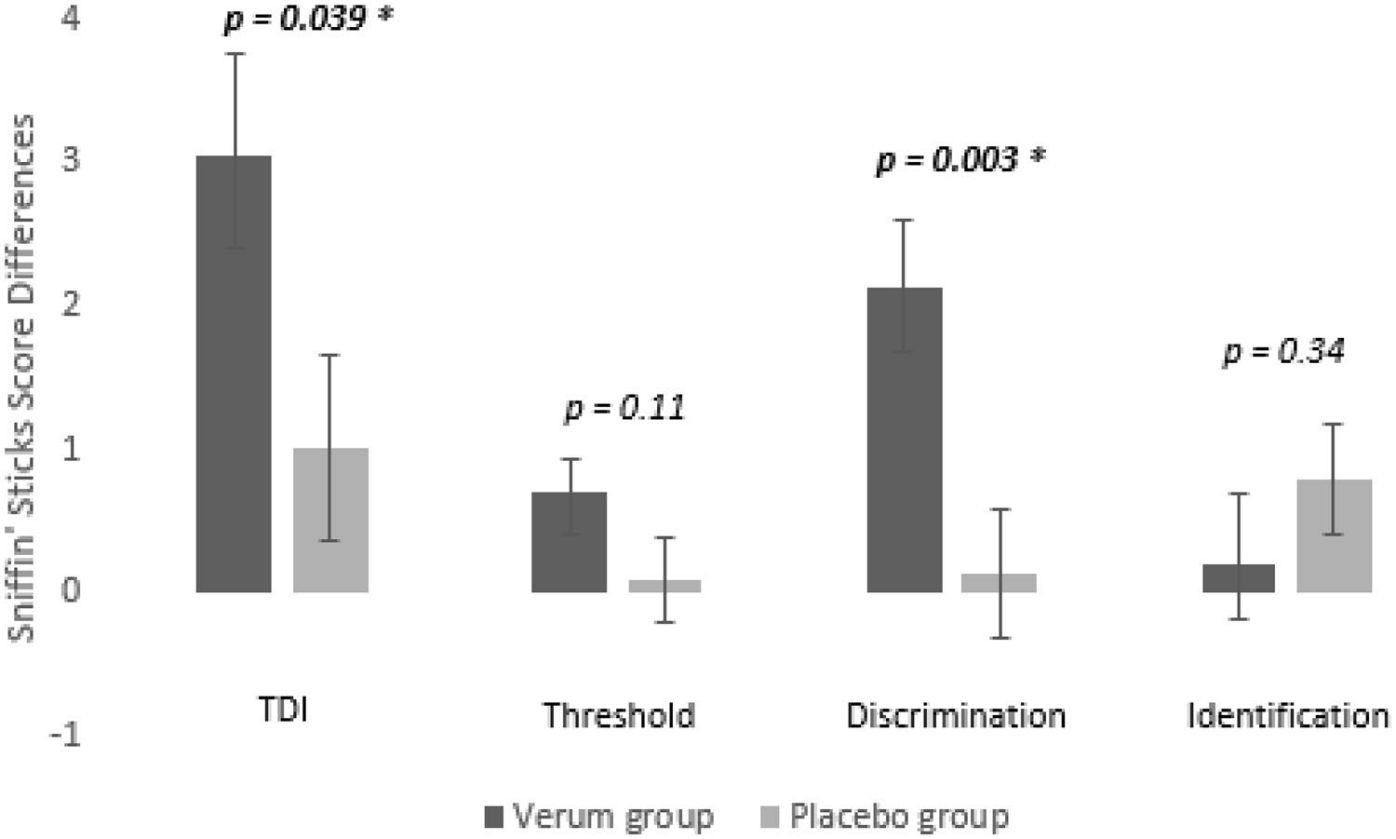
Mean differences (scores after treatment minus scores before acupuncture) of olfactory test results with Standard Errors; * shows significant results

When comparing the change of TDI scores before and after treatment the verum group performed better than the sham acupuncture group (F = 4.45, *p* = 0.04) Concerning individual subtests, only odour discrimination was significantly different between the two groups (F = 9.48, *p* = 0.003), but not odour threshold (F = 2.61, *p* = 0.11) and odour identification (F = 0.93, *p* = 0.34) These results can be seen in Fig 1 With regard to a clinically significant improvement on an individual level, 6 of 30 subjects from the verum group (20%) exhibited improvement of more than 5.5 points in the TDI score, whereas only 3 of 30 subjects exhibited improvement in the placebo group (10%) [39]. While this is not a statistically significant result, it does deserve mention.

**Fig 2:**
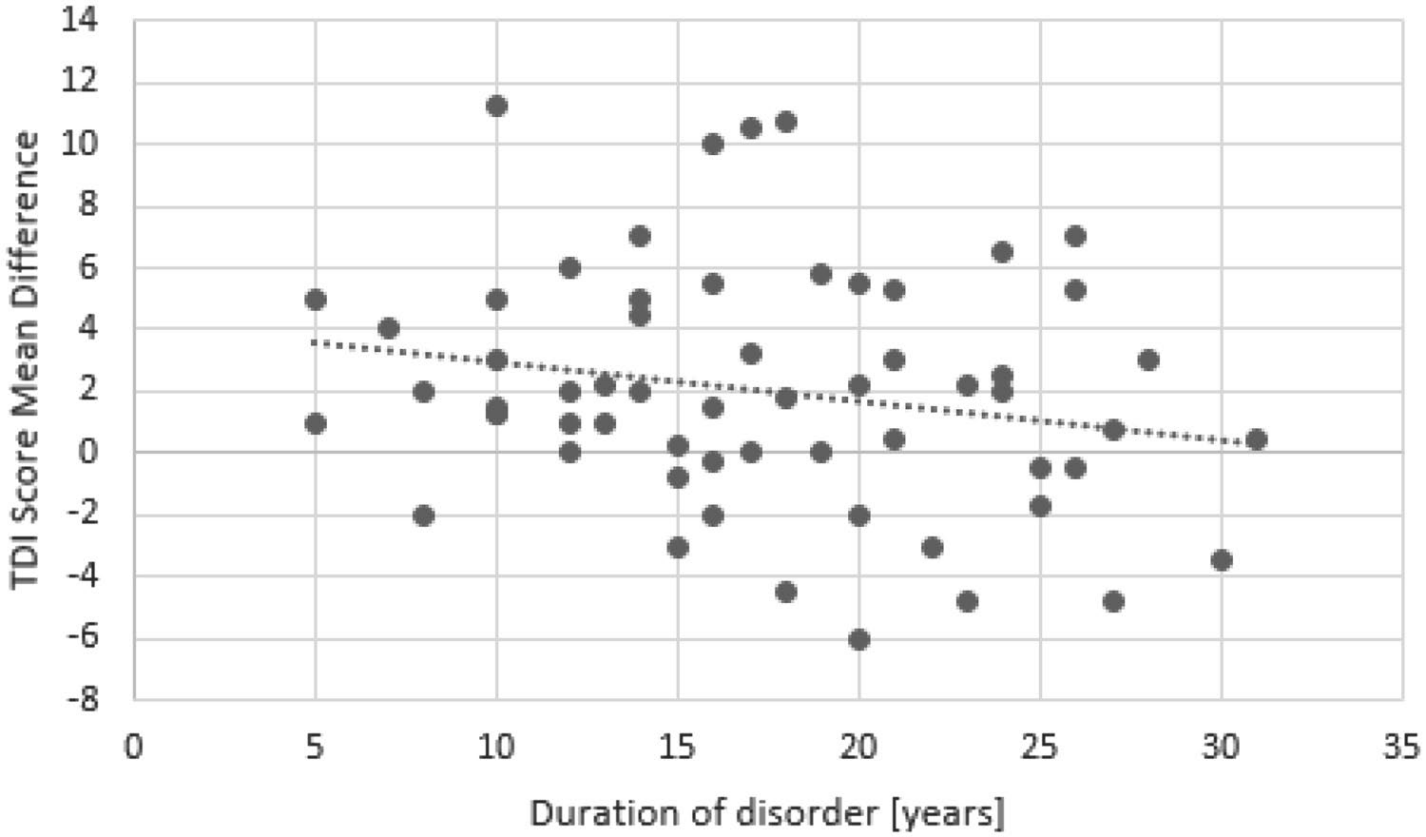
Correlation between changes in TDI scores and duration of smell loss

When analysing data across both groups there was a significant correlation between the change of TDI scores and the duration of the disease (r = − 0.4, *p* = 0.001) with better outcome in patients with a shorter duration of the olfactory loss (Fig 2).

## Discussion

The present results indicated that acupuncture has a positive effect on olfactory function in patients with postinfectious smell loss We found a significant improvement in general olfactory performance as well as an improvement in odour discrimination in verum acupuncture compared to sham treatment On an individual level, verum acupuncture increased olfactory function in 20% of the subjects compared to 10% of subjects who had sham acupuncture Further, the treatment response correlated negatively with the duration of smell loss—the longer the smell loss the less likely it was to achieve a positive treatment response A similar relation between the duration of olfactory loss and improvement of olfactory function has been reported several times [15, 40]. Acupuncture appeared to be useful independently from the patients´ age.

The results of our study confirm some of the findings of previous studies. The present research tried to avoid several issues that limited the significance of previous studies, e.g., lack of a control group, heterogeneous patient groups, or low case numbers [30, 32–34]. We, therefore, included a homogenous, thoroughly diagnosed patient group with postinfectious smell loss, and a patient-blinded control condition to minimize possible bias. The therapy was well accepted by the patients, which can be seen from the lack of dropouts and the absence of side effects.

It is interesting to note that odour discrimination, but not odour threshold, improved in response to acupuncture An explanation could be that odour discrimination appears to involve higher-level cognitive functions to a higher degree compared to odour thresholds [41]. Acupuncture has previously been shown to have effects on cognitive function tested with the Mini-Mental State Examination Test in patients following a stroke [42, 43]. Therefore, it might be hypothesized that acupuncture has positive effects on the cognitive processing of odours For example, the outcome of the test might have been positively modified by different levels of attention and concentration.

Acupuncture is an important part of traditional Chinese medicine When analysing this form of treatment, it is important to remember that the classical scientific proof is difficult to obtain when the foundation of the treatment includes something that evades measurement It is, however, possible to focus on the effects of the treatment, which is what was done in this study.

## Conclusion

The present results suggest that acupuncture is helpful in patients with postinfectious olfactory loss. The shorter the time span between smell loss and treatment, the more likely it is for the treatment to have a positive effect.

With hardly any negative side effects being described, acupuncture should be considered as a supplementary treatment.

Future studies need to determine whether the observed increase of olfactory sensitivity is temporary or is lasting for a longer period of time.
